# Coarse-Grained Models Reveal Functional Dynamics – II. Molecular Dynamics Simulation at the Coarse-Grained Level – Theories and Biological Applications

**DOI:** 10.4137/bbi.s459

**Published:** 2008-03-05

**Authors:** Choon-Peng Chng, Lee-Wei Yang

**Affiliations:** Institute of Molecular and Cellular Biosciences, University of Tokyo, Tokyo 113-0032, Japan

**Keywords:** conformational transitions, molecular mechanics, Gō-model, Boltzmann-Inversion, simulation time-step

## Abstract

Molecular dynamics (MD) simulation has remained the most indispensable tool in studying equilibrium/non-equilibrium conformational dynamics since its advent 30 years ago. With advances in spectroscopy accompanying solved biocomplexes in growing sizes, sampling their dynamics that occur at biologically interesting spatial/temporal scales becomes computationally intractable; this motivated the use of coarse-grained (CG) approaches. CG-MD models are used to study folding and conformational transitions in reduced resolution and can employ enlarged time steps due to the absence of some of the fastest motions in the system. The Boltzmann-Inversion technique, heavily used in parameterizing these models, provides a smoothed-out effective potential on which molecular conformation evolves at a faster pace thus stretching simulations into tens of microseconds. As a result, a complete catalytic cycle of HIV-1 protease or the assembly of lipid-protein mixtures could be investigated by CG-MD to gain biological insights. In this review, we survey the theories developed in recent years, which are categorized into Folding-based and Molecular-Mechanics-based. In addition, physical bases in the selection of CG beads/time-step, the choice of effective potentials, representation of solvent, and restoration of molecular representations back to their atomic details are systematically discussed.

## Introduction

Atomistic-MD simulation for biomolecules in aqueous medium has shown great success in reproducing experimentally observed dynamical events that occur at time-scales of tens to hundreds of nanoseconds. However, the technique inevitably meets its limit when the size of the system under study gets large (> few hundred residues for proteins) or the biochemical processes in question occur at a longer time-scale. It is currently not computationally tractable to follow the course of protein folding/unfolding (taking microseconds to seconds, the time regime of greatest biological interest) or disease-related protein association/aggregation processes that could take even longer with atomistic-MD. Furthermore, a single simulation run suffers from insufficient sampling of the dynamics of interest leading to inconclusive results, hence the need for faster sampling approaches to acquire adequate statistics.

**Figure 1:**
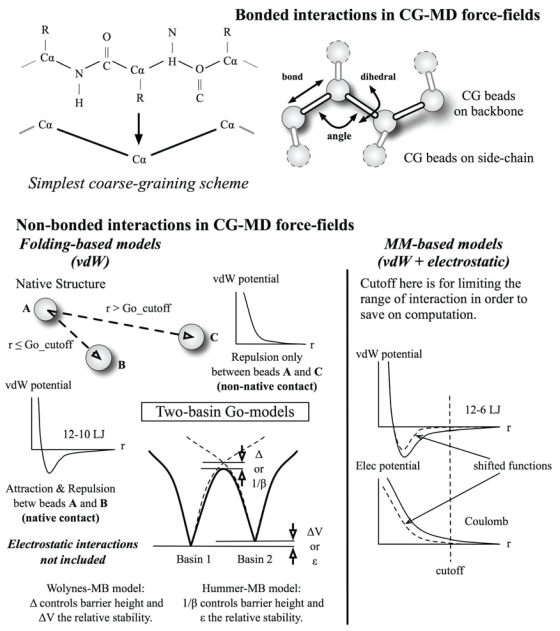
Interactions between CG-beads can be broadly divided into ‘bonded’ (bond, angle, dihedral) and non-bonded. For MM-based models, non-bonded interactions include vdW and electrostatics which are commonly represented by 12-6 LJ and Coulomb potentials, respectively. For Folding-based models, pairs of beads in native contact (separation within a cutoff of about 8 Å) in the reference structure are under vdW interactions (commonly represented by 12-10 LJ) while those not in native contact repel from each other. The folding (free) energy surface for each protein structure can be described by a smooth funnel-shaped basin with the native conformation having the minimum energy at the bottom of the basin. These basins can be connected with adjustable relative stability and barrier height to study conformational transitions.

To overcome temporal, spatial and statistical limitations with reduced computation cost, a coarse-grained (CG) strategy has been suggested to represent molecules with groups of atoms (the pseudo-atoms or beads) that interact via effective force-fields (Fig. 1). The idea dates back to the 70s with pioneering work by Levitt and Warshel (Levitt and Warshel, 1975; Levitt, 1976) where backbone nitrogen, carbon and hydrogen atoms are grouped into a bead in studies of protein folding. Residue side-chain is represented by one bead at the center-of-mass. Replacement of groups of atoms by CG beads essentially averages out the all-atom dynamics causing a smoother interaction potential and allows the use of large simulation time-steps without introducing numerical instabilities. This is analogous to the allowed increase of the typical atomistic-MD time-step from 1-fs to 2-fs when the fastest motions involving hydrogen atoms are removed. Current CG models can extend the practicality of MD simulations from ~100-ns to ~10-μs, a factor of 100 (Arkhipov et al. 2006a; Bond and Sansom, 2006; Shih et al 2007; Trylska et al. 2007).

In this review, we emphasize on CG models developed for MD simulations of proteins and briefly mention lipid models that are comprehensively reviewed by Venturoli (Venturoli et al. 2006). CG-EN models introduced in part I of this review series, despite successes in predicting deformations that agree with experimental results, are not formulated to describe large amplitude conformational changes accompanied by partial unfolding (breaking and forming of residue contacts) and cannot simulate assembly processes of macromolecules such as proteins or lipids.

### Overview of CG-MD models

Current CG-MD methods can be broadly divided into two categories based on how non-bonded interactions (usually separation by more than three bonds) between pseudo-atoms are treated (Fig. 1 lower panel). Many CG-MD models employ energy terms between beads which are inspired by atomistic Molecular Mechanics (MM) forcefields. These will be referred to as *MM-based* models (see Table 1). One such model has enabled the simulation of the full catalytic cycle of Human Immunodeficiency Virus type 1 protease (HIV-1 PR) (Trylska et al. 2007). HIV-1 PR is a crucial enzyme in the life cycle of the AIDS causing virus, cleaving nascent polyproteins into functional protein products that are essential for viral assembly and subsequent activities during viral replication. Due to rapid mutations of the enzyme, which impair the possible competitive inhibition of therapeutic compounds at the active site, an intervention strategy has been reformulated to target the enzyme dynamics instead. A proposed view of the entire enzymatic cycle from substrate entry to exit was presented by Trylska based on microsecond-long simulations using their CG-MD model (Trylska et al. 2007).

Simplified protein models inspired from protein folding studies also allow simulation of large-scale conformational transitions between observed states, termed *Folding-based* models (see Table 2). In the Gō-like models (Taketomi et al. 1975) of proteins, the potential energy of a protein conformation lies on a funnel-shaped basin with the lowest-energy structure at the bottom being the experimentally determined ligand-bound or unbound (free) conformer. Also, only residue pairs within a certain cutoff distance in a determined structure contribute favorable non-bonded interactions to the total energy. Different approaches have been taken to smoothly ‘fuse’ such basins in order to create multi-basin CG-MD models that describe changes from one state to another, which typically take microseconds or longer (Okazaki et al. 2006; Best et al. 2005). Okazaki formulated the task of merging potentials as an eigenvalue problem (similar approach as taken for the Plastic Network Model by Maragakis and Karplus, 2005, introduced in our accompanying review on CG-ENM). The resultant model was used to study transitions between ligand-free and ligand-bound structures, where local unfolding has been shown to facilitate such processes (Okazaki et al. 2006). This model can be viewed as an improvement over the ‘switching Gō-model’ proposed by Koga and Takada where the single basins for each F1-ATPase nucleotide-bound state are not smoothly connected. Transitions triggered by sudden switch of energy basins upon ATP-binding are then followed by a period for system relaxation to the new minimum (Koga and Takada, 2006).

An alternative approach to building multi-basin models taken by Hummer and co-workers was to add up the potentials via a Boltzmann-weighting procedure (Best et al. 2005; Chen and Hummer, 2007). This approach is physically intuitive and adaptable such that a combination of multiple ENM’s elastic potentials (Zheng et al. 2007) or all-atom detailed potentials is possible. Conformational dynamics and unfolding of the calmodulin C-terminal domains were investigated using this model (Chen and Hummer, 2007). Despite the absence of calcium, each of the two domains of calmodulin is known to change from the apo/folded (‘closed’) state to the hydrophobic-core-exposed holo-like (‘open’) state at NMR-characterized μs to ms time-scale, and this transition is successfully captured by the multi-basin CG-MD model at 300 K. A third apo/unfolded state found in the T-jump experiment appears in the CG-MD simulation at the melting temperature of 320 K. This state together with the apo/folded state defines the two-state folding transition.

Details of the two multi-basin models above will be given in the THEORY section. Note that in such multi-basin methods, the frequency of occurring transitions is controllable by adjusting barrier heights and relative stability parameters, as opposed to the incidentals observed in atomistic-MD or, to some extent, MM-based CG-MD simulations.

Various supramolecular assemblies of biomolecules are of great biological interest, such as molecular machines like the ribosome and dynamic structures like the bacterial flagella and virus capsid. Due to their large sizes (diameters of 100–1000 Å) only nanosecond dynamics of the smallest virus capsid could be studied by atomistic-MD (Freddolino et al. 2006). MM-based models developed based on an assignment scheme of CG-beads to preserve the shape of capsid subunits have allowed microsecond-long simulations of empty and filled capsid with diameters ranging from 160 to 750 Å (Arkhipov et al. 2006a; see also Schulten-SA model in Table 1), affording insights into their conformational stability. Parameterization of the effective potentials between beads was performed by Boltzmann-Inversion (BI) procedure (see the THEORY section) based on statistical distribution accumulated from short (<10-ns) atomistic-MD simulations in explicit solvent of atomic clusters about the size of a CG bead.

The coarse-graining of actin filament monomers also involves use of atomistic-MD for parameterization (Chu and Voth, 2006). Each monomer model contains 4 beads, each of which represents a subdomain. Within a monomer, beads interact with each other via potentials consisting of three bond-stretching terms, two angle-bending terms and one dihedral term (total of six degrees of freedom). Between monomers, beads interact via ENM potentials (see our companion article on CG-ENMs). The force constants of ENM bonds between monomers together with those of the six harmonic interactions in each monomer are adjusted to best-fit the fluctuation profiles between those obtained from ENM and from a 10-ns atomistic-MD trajectory using an iterative procedure, named *fluctuation-matching* (Chu and Voth, 2006).

Current studies of μsec or longer lipid-protein assembly process are hindered by the limit of ~100-ns dynamics sampled by atomistic-MD. CG-MD models are well positioned to address such deficiency. MM-based CG models developed for efficient and accurate simulations of lipid systems (Marrink et al. 2004, 2007) have been extended to incorporate amino-acid residues to study such formation of lipid bilayers with embedded membrane proteins (Bond and Sansom, 2006) and to understand how amphipathic proteins can act as scaffolds to hold lipid bilayers together in a nanodisc, the mimic of a nascent high-density-lipoprotein particle (Shih et al. 2006, 2007). Notably, each residue is represented by one bead for backbone and up to two beads for side-chain, with these beads being classified into four polarity types. The aforementioned models are to be further illustrated in the THEORY section.

Combination of a step-function approximation to continuous bonded and non-bonded (Folding-based) potentials with collision-based molecular dynamics represents a more aggressive approach to coarse-graining (Ding and Dokholyan, 2005). The authors claimed that such discrete MD (DMD) could be up to 10 billion times faster than atomistic, continuous MD! Each DMD time-unit was estimated to correspond to about 0.5-ns of physical time, implying that 100,000 time-units span roughly 0.5-μs. Release of vinculin auto-inhibition (Chen and Dokholyan, 2006) and μs dynamics of nucleosome core particles (NCP) (Sharma et al. 2007) were successfully studied with this method. However, the very simplified models come at the price of a huge loss of interaction details. As a result, they’re good for studying macromolecular assembly but not detailed chemical interactions.

How to obtain accurate and if possible, transferable parameters for the CG force-fields is still under development (Tozzini, 2005). In the THEORY section, we illustrate the popular Boltzmann-Inversion parameterization scheme in detail using one MM-based model as an example. In the DISCUSSION section we highlight the current trends and issues which we reckon among the most important to be addressed for CG-MD simulations to provide clear physical insights into biological systems.

## Theory

### Molecular mechanics-based CG-MD models

In the Molecular Mechanics models, van der Waals (vdW) and electrostatic interactions between atoms are generalized to the pseudo-atoms (illustrated in Fig. 1 lower right). These simplified models contain interactions between CG beads analogous to potentials found in typical molecular mechanics force-fields such as CHARMM (Brooks et al. 1983) or AMBER (Case et al. 2004). As these models are simply extensions of atomistic-MD, existing all-atom simulation packages can readily be used with minimal changes. Unlike the Folding-based models (introduced later), there is no bias towards a reference structure as both native and non-native contacts are included. Various MM-based CG-MD models are summarized in Table 1.

**Figure 2:**
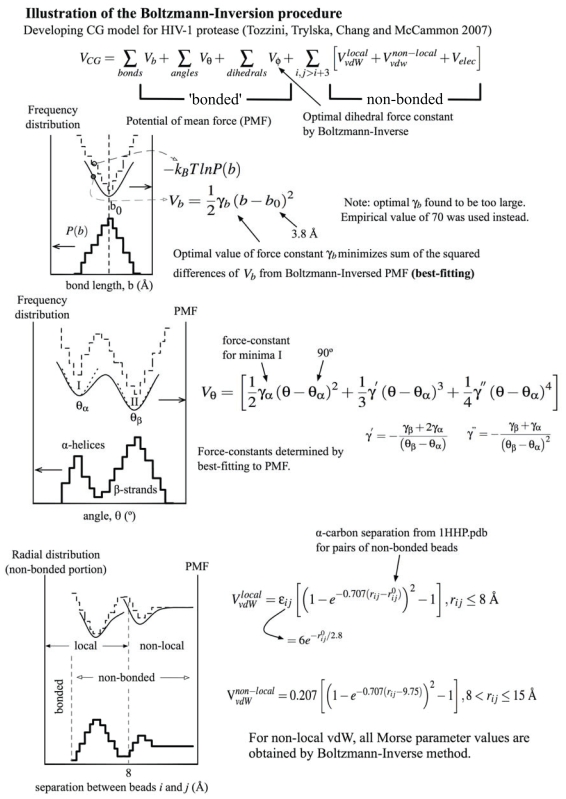
Parameterization of the force-field of the TTC model of HIV-1 protease, based on the Boltzmann-Inversion procedure. The distribution for angles involved in β-strands is bi-modal but is single-modal when specific residue sequences are considered. With θ_α_ and θ_β_ determined, force constants are optimized via least-squares-fit procedure. Dihedral potential is assumed harmonic, with the force constants taken from BI but equilibrium dihedral angles taken from the reference structure. Lastly, for the non-bonded vdW interactions, there is one PMF well for the non-local part (>8 Å) but multiple ones, of which equilibrium distances are taken from pair separations (*r_ij_*^0^) of beads in the reference structure, for the local part (within 8 Å).

In analogy to atomistic-MD with an empirically defined molecular mechanics force-field with parameters optimized to reproduce experimental observations or quantum mechanical calculations, we can assign CG-MD force-field parameters to either reproduce properties observed from atomistic-MD or from a statistical analysis of known protein structures deposited in the PDB. One popular scheme is the Boltzmann-Inversion (BI) method (Reith, 2003). We assume that the ‘inverse’ of the Boltzmann probability distribution is a good first approximation to the potential energy (strictly speaking, this should be the free energy but the entropy component is often assumed to be constant) given as: *V(q)* = −*k_B_T* ln(*P(q)*). Here, *q* is some internal coordinate such as angle or separation of CG beads, *k_B_* is the Boltzmann factor, *T* is the absolute temperature, *V(q)* and *P(q)* are the potential-of-mean-force (PMF) and the probability/frequency distribution at *q*, respectively. *P(q)* can be obtained from either accumulative counts derived in a short atomistic-MD simulation data or a statistical analysis of experimental structures from the PDB. Since *P(q)* is often a discrete distribution, the BI potential is only discrete and has to be best-fitted by continuous functions. See Figure 2 for an illustration of the BI parameterization of the TTC model for HIV-1 PR.

#### Model for HIV-1 protease enzyme dynamics

The HIV-1 PR is a 198-residue homodimer, with access to the active site controlled by two flexible β-hairpins known as ‘flaps’. To simulate the complete process of substrate entry, stabilization and exit after peptide-bond cleavage which involves flap opening/closing events that take microseconds or longer, we have to resort to a simplified representation of the enzyme and substrate.

The TTC model (short for Trylska-Tozzini-McCammon; Table 1) developed for modeling the HIV-1 PR consists only of alpha-carbon CG beads that interact via pseudo-bond, -angle and -dihedral ‘bonded’ potentials as well as vdW and electrostatic non-bonded interactions (Tozzini and McCammon, 2005, Chang et al. 2006). For the crucial task of determining the force-field parameters, the BI procedure was used (see Fig. 2, adapted from Supplementary Information to Tozzini et al. 2007). To build up the frequency distributions, a statistical set of experimental structures was created based on a collection of 100 known HIV-1 PR structures and 100 randomly-chosen *α*-helical proteins. For ‘bonded’ interactions, the pseudo-bond and pseudo-dihedral potentials are assumed harmonic. In practice, no differences in the results were found if bonds were simply fixed to their equilibrium values (Tozzini and McCammon, 2005). For each set of 1–4 beads, the equilibrium dihedral value was taken from a reference structure (PDB code 1HHP), while the associated harmonic force constant is optimized using BI procedure. Emphasis was placed on the angle term, where the PMF has one minimum for angles involved in α-helices but two minima for those in *β*-strands. However, when taking the residue identity into consideration, the angles in *β*-strands follow single-modal distributions and therefore produce one minimum for the PMF. Thus, the statistical potential can be fitted with two separate harmonic wells or a single *quartic* double-well potential (Fig. 2). Only the quartic form allows the *β* to *α* transition that is crucial for flap curling, the prerequisite step for flap opening (Tozzini and McCammon, 2005). This is an example where CG force-fields have to be tuned for a particular system of interest.

For charge-charge interactions, a screened Coulomb potential with a distance-dependent dielectric constant was used. Integer charges were assigned to ionizable residues according to their protonation state at physiological pH. In the case of vdW interactions, C_α_-based radial distribution function (a reflection of local order) (Leach AR, 2001) constructed from the statistical dataset with ‘bonded’ atoms excluded was used to derive the PMF. The two resulting energy minima regions representing local and non-local non-bonded contributions to the vdW term could be separated by a cutoff distance of 8 Å and hence fitted separately with Morse potential functions (Fig. 2; Tozzini and McCammon, 2005). The scheme herein only produced a weak bias to the reference structure since native non-bonded interactions can be broken and new non-native ones formed, in contrast to Folding-based models where non-native contacts are unfavorable.

What has simulations using this CG-MD model unraveled about HIV-1 PR enzyme dynamics? NMR experiments have indicated that flap movements occur over micro- to milli-seconds with faster motions in the sub-nanoseconds (Freedberg et al. 2002). In their first application of the model, Tozzini and McCammon reported that flap opening/closing events during a 10-μs simulation trajectory exhibit ‘multiple frequency dynamics’. Peaks appearing in the time variation of flap-tip distances (indicating opening/closing events) are spaced at short time intervals of 5 to 10-ns giving a grouped appearance, representing the high-frequency dynamics. These groups of peaks are spaced on longer time intervals of ~100-ns, representing the low-frequency dynamics. All these events were observed on the microsecond-long simulation time-course (Tozzini and McCammon, 2005). This might explain why opening/closing events can sometimes be captured in atomistic MD of <100-ns duration (Hornak et al. 2006) but not always. Tozzini also found that flaps can vary from closed to semi-open, open and wide-open configurations during a 10-μs CG-MD simulation (Tozzini et al. 2007). Due to crystal-packing constraints, open and wide-open structures (flap tip separations of 20 Å or more) might not be observable in X-ray structures. Furthermore, exploration for favorable substrate orientation in order to enter the binding site takes around 1-μs. After the cleavage of substrate peptide-bond (simulated by adjustments to substrate CG force-field), products left the active site on the nanosecond time-scale but remained close to the enzyme surface and diffuse away only after about 0.5-μs (Trylska et al. 2007). Such a detailed view of the complete catalytic cycle of HIV-1 PR could prove useful in the design of therapeutic agents against HIV-1.

#### Models for realistic lipid-protein assembly studies

The two models about to be introduced here (see Table 1) are extensions of a CG lipid model by Marrink (Marrink et al. 2004), who proposed an improved model over Shelley’s parameterized lipid model (Shelley et al. 2001). In the Marrink model, four water molecules are combined into one polar CG water bead (type P). Polar beads are also used to represent lipid head-groups while lipid tails require hydrophobic beads (type C). Nonpolar beads (mixed polar and hydrophobic, type N) and charged beads (type Q) are also defined. For example, a DPPC lipid molecule is composed of Q, N and C beads. Beads of types N and Q are further divided into subtypes {d, a, da, 0} depending on whether they can be H-bond donors (d), acceptors (a), both or neither. Beads interact through harmonic pseudo-bond, pseudo-angle terms, non-bonded vdW (using LJ potential) and electrostatic (using screened-Coulomb potential) forces. Besides faster than Shelley’s, the Marrink model is not tailored for any specific lipid phase and reproduces various experimentally measured structural, elastic and thermodynamics properties (Marrink et al. 2004). Model parameters are optimized using atomistic simulation data and an overall speedup of 3–4 orders of magnitude as compared to atomistic-MD is reported. Simulated events occur four times faster than in experiments (Marrink et al. 2004, 2007).

The lipid-protein CG model from Sansom group (Sansom-LP model in Table 1) uses one N bead to represent the four protein backbone atoms (with subtype depending on the presence of H-bonds in starting atomic structure) and up to two beads for the side-chains (Bond and Sansom, 2006). Following the Marrink’s scheme for bead type assignment, a Lys/Arg side-chain is represented by C+Qd beads while a Phe has C+C beads. Secondary structural elements are maintained via extra harmonic distance restraints imposed on non-bonded beads (Table 1). This lipid-protein model was used to study assembly of lipid bilayers in the presence of membrane proteins. A 5-μs simulation was performed using the GROMACS package (Lindahl et al. 2001) with a 40-fs time-step.

The model from Schulten group (Schulten-LP model in Table 1) uses one Nda backbone bead for all residues and only one side-chain bead at the center of mass regardless of the size of side-chain (Shih et al. 2006). Also, a cosine-series pseudo-dihedral term is included. Other settings are the same as Sansom’s. The CG model accelerates the simulation by 1500 folds as compared to atomistic-MD: dynamics progress 50–150 times faster with the larger time-step sizes of 25 to 50-fs plus a 10 times reduction in the degrees of freedom, which results in faster relaxation processes. The model was used in studying the assembly of nanodiscs: two amphipathic helical proteins (apolipoprotein A-I or apo A-I) wrapping a lipid bilayer patch. Preassembled CG-models of double-belt nanodiscs remained stable over periods of around 100-ns. However, disordered-fence configurations rather than the double-belt configurations were observed at the end of assembly simulations lasting about 1 to 1.5-μs (Shih et al. 2006). Use of an improved scheme where the dihedral parameters were re-parameterized via BI based on statistics from an atomistic MD simulation of a *α*-helical segment of apo A-I in order to preserve the secondary structure produced a configuration resembling the double-belt in 4-μs simulation (Shih et al. 2007).

### Folding-based CG-MD models

#### Gō-like models for protein folding

This is an off-lattice model that typically contains beads only at alpha-carbon positions to represent residues. The interactions between beads are encoded with energetic preference towards the native structure. For non-bonded vdW, favorable interactions between residues in contact (within a cutoff distance) in the native state and purely repulsive ones between non-native contacts are defined (illustrated in Fig. 1 lower panel). Two popular versions of the Gō-model have emerged: the Clementi-Onuchic model (Clementi et al. 2000) and the Karanicolas-Brooks model (Karanicolas and Brooks, III 2002). They formed the basis for multi-basin models introduced below. Table 2 presents a comparison of energy terms employed in each model.

##### Clementi-Onuchic model

In this formulation, pseudo-bond and pseudo-angle potentials for the C_α_ CG beads are taken as harmonic, while a two-term cosine-series is used for the pseudo-dihedral potential (Clementi et al. 2000),

$$\begin{array}{l} \sum_{dihedral}\gamma_{\varphi }^{\left(1\right)}\left[1+\text{cos}(\varphi -\varphi_{0})\right]\\ +\gamma_{\varphi }^{\left(3\right)}\left[1+\text{cos}3(\varphi -\varphi_{0})\right] \end{array}$$

CG beads separated by more than three pseudo-bonds are also involved in non-bonded interactions. Van der Waals interactions between native and non-native pairs of beads separated by a distance *r_ij_* are:

$$\begin{array}{c} vdW_{native}(r_{ij})=ɛ(i,j)[5{\left(\frac{\sigma_{ij}}{r_{ij}}\right)}^{12}-6{\left(\frac{\sigma_{ij}}{r_{ij}}\right)}^{10},\\ vdW_{non-native}(r_{ij})={ɛ}_{2}(i,j){\left(\frac{\sigma_{ij}}{r_{ij}}\right)}^{12} \end{array}$$

Well depths *ɛ*(*i,j*) and *ɛ*_2_(*i,j*) are tunable constants. Collision diameters *σ_ij_* are taken as alpha-carbon separations of residues *i* and *j* in the native structure for the native pairs and equal to 4 Å for non-native pairs (Clementi et al. 2000). Force constants *γ* for bond, angle and dihedral terms are chosen such that interaction energy contributed by these torsional degrees of freedom is only about half the non-bonded (by tertiary contacts) energy that is the primary driving force for folding cooperativity (Clementi et al. 2000). In the recent minimalist model from Clementi group, the optimal values of *ɛ* for native pairs are chosen to satisfy a set of experimentally measured free energy changes upon mutation (Matysiak and Clementi, 2004). The Das, Matysiak and Clementi (DMC) model that retains nonnative interactions by incorporating residue-type information in the iterative refinement of vdW parameters represents an attempt to go beyond the standard Gō-like model (Clementi 2007).

##### Karanicolas-Brooks model

This model differs from the Clementi-Onuchic model mainly in the use of backbone-backbone and side-chain-side-chain hydrogen-bond assignments to define native contacts between residues, with H-bonds assigned according to an energy threshold. The vdW interaction between native residue pairs is a 12-10-6 modified LJ potential that incorporates a low energy barrier (termed desolvation penalty) before interaction becomes attractive as the pair of beads comes together from initially being far apart (say in the unfolded state),

$$\begin{array}{l} vdW_{native}(r_{ij})=ɛ(i,j)[13{\left(\frac{\sigma_{ij}}{r_{ij}}\right)}^{12}\\ -18{\left(\frac{\sigma_{ij}}{r_{ij}}\right)}^{10}+4{\left(\frac{\sigma_{ij}}{r_{ij}}\right)}^{6} \end{array}$$

Here, *ɛ*(*i,j*) is also the interaction strength but varies for each pair of contact. This functional form for vdW shows an enhanced folding cooperativity of 2-state folders (those proteins lacking folding intermediate(s) between unfolded and folded states). Residues H-bonded in the native structure interact via the above vdW potential with *ɛ*(*i,j*) = 1 and *σ*_ij_ being the alpha-carbon separation. Non-native residue pairs repel with a 1*/r*^12^ potential identical to that used in Clementi-Onuchic model. Beads separated by three or more pseudo-bonds (in contrast to four or more in the Clementi-Onuchic model) are included in the vdW contacts in order to represent explicitly the backbone H-bonds between residues in *α*-helices. To maintain backbone orientation between *β*-strands, four additional weak native contacts each with *ɛ*(*i,j*) = 0.25 were defined for residue pairs in the neighborhood of an H-bonded pair. For residues interacting via side-chains, each pair adopts different LJ energy parameters to reflect the differences in side-chain chemistry. Lastly, the dihedral potential was obtained by a BI procedure on a distribution derived from Ramachandran plots of the 20 × 20 pairs of ordered amino-acid residues collected from PDB structures. The resulting potential is sequence-specific (but not topology-specific) with two minima, centered at α-helical and β-strand geometries. A four-term cosine-series is best-fitted to this statistical potential.

### Multiple-basin models for large-amplitude conformational changes

Many proteins undergo a large conformational change between ligand-free and ligand-bound states (Sun et al. 1998; Ishima et al. 1999). In order to describe such a change from one equilibrium conformation to another, a ‘fusion’ of respective energy basins into a continuous energy surface is needed (a schematic double-basin potential is illustrated in lower left panel of Fig. 1). Two such models have been proposed with promising extensions to multiple basins (to include intermediate conformations).

The Takada-Onuchic-Wolynes (T.O.W.) research groups jointly proposed a model (we termed it as Wolynes-MB; see Table 2) where two or more Clementi-Onuchi Gō-potentials representing native structures are combined smoothly (Okazaki et al. 2006). A pair of residues is taken to be in the native contact if the smallest inter-residue separation for non-hydrogen atom pairs is less than 6.5 Å. During conformational transition between basins, developed strains in the system are relieved by local unfolding that is facilitated with use of ‘softened’ angle and dihedral force-constants. If harmonic strain energy (in terms of angle or dihedral) between the two conformational states becomes larger than a predefined cutoff, the new force-constant is taken to be the ratio of the cutoff energy to strain energy. For a two-basin model, the energy barrier and relative stability can be adjusted via parameters Δ and Δ*V* as follows: increasing Δ would lower the barrier height while increasing Δ*V* makes one minima higher than the other (See Fig. 1). The smoothly connected multi-basin potential *V_MB_*,

$$\begin{array}{l} V_{MB}=\frac{V(R|R_{1})+V(R|R_{2})+\Delta V}{2}\\ -\sqrt{{\left(\frac{V(R|R_{1})-V(R|R_{2})-\Delta V}{2}\right)}^{2}+\Delta^{2}} \end{array}$$

is mathematically formulated as the smallest eigenvalue of the matrix C (Okazaki et al. 2006), where *V*(*R*|*R_i_*) is the energy of the evolving conformation *R* in the basin of which equilibrium/native conformation *R_i_* sits on the bottom.

$$C=\left(\begin{array}{cc} V(R|R_{1}) & \Delta \\ \Delta & V(R|R_{2})+\Delta V \end{array}\right)$$

Employing the Wolynes-MB model, conformational transitions of four proteins (Glutamine-Binding-Protein, HIV-1 PR, dihydrofolate reductase and a structural analog of calmodulin) between their respective unligated (*open*) and ligated (*closed*) forms were simulated, involving hinge and shear domain motions. The barrier height was decreased by increasing Δ until frequent visits between two basins could be observed. The relative stability parameter Δ*V* was also adjusted to give equally frequent transitions between basins. Separate sets of values were used for each of the four proteins. Simulations were performed at the optimal temperature of 0.8 *T_f_^min^*, which is the smaller of the folding temperatures of the two protein structures. The authors reported sudden and infrequent transitions between energy basins, with signatures of local unfolding. The Wolynes-MB model could in principle extended to any number of basins, which involves repetitively solving the 2 × 2 aforementioned matrix for the combined potential of every two energetically adjacent wells.

In the Hummer-MB model (Table 2) single-basin Karanicolas-Brooks Gō-potentials are merged via summing up the corresponding Boltzmann weights (see below) (Best et al. 2005). This is physically equivalent to pooling together all accessible conformational sub-states defined at each energy level. The combined potential for a *M*-basin model has the expression,

$$\text{exp}(-\beta V_{MB})=\sum_{i=1}^{M}\text{exp}\left(-\beta \left(V(R|R_{i})+{ɛ}_{i}\right)\right)$$

where *β* = 1/*k_B_T* and parameter *ɛ_i_* controls the relative stability of the separate energy basins (Best et al. 2005). As suggested from the above expression, this model can be extended to use ENM’s harmonic (Zheng et al. 2007) or all-atom transferable potentials as *V(R*|*R_i_)*.

The Hummer-MB model was first applied in the investigation of the beta-sheet (as in the wild-type structure) to 3_10_-helix (as in the N11L-L12N double-mutant structure) transition of the N-terminus domain (*switch* region) of dimeric Arc Repressor. This transition was observed to occur in the N11L mutant on micro- to milliseconds by NMR, necessitating a CG treatment. Such secondary-structural changes could not be captured by single-basin elastic network or Gō models (Best et al. 2005). To allow for such a *switch*, the pseudo-angle term takes on a sequence-independent (in contrast to MM-based TTC model which takes residue-identity into account) *double-well* potential for residues in *α* and *β* structures as derived by BI method on a set of 500 PDB structures. Lengths of pseudo-bonds between C_α_ beads were fixed at the average values of the two structures. Parameter *ɛ_i_* for each Gō model was set to zero. Contacts for *non-switch* region were taken from sheet structure (though equivalent to helix structure) while those involved with *switch* region residues are taken from sheet or helix structures. Conformational transitions (local unfolding and rapid refolding of the *switch* region) were identified during 6-μs simulations. The model was subsequently modified: simpler 12–10 potential replaced the 12-10-6 potential; the combined potential energy is defined through exp(−*β*′*V*) = exp(−*β*′*V_apo_*) + exp(−*β*′(*V_holo_*+*ɛ*)) with (1/*β*′) not *k_B_T* but implying the barrier height (Chen and Hummer, 2007). These parameters (1/*β*′) and *ɛ* serve similar roles as Δ and Δ*V* in the Wolynes-MB model, respectively. In their calmodulin work, Chen chose values for *β*′ and *ɛ* to reproduce experimentally characterized rates of conformational transitions between *apo* and *holo* forms (Chen and Hummer, 2007).

The above studies employing multi-basin Gō-models supported the notion raised by the T.O.W. groups that localized unfolding facilitated large-scale ligand-induced conformational changes. Such accompanied partial unfolding cannot be accessed by the CG-EN models, even with the use of the PNM model (see Supplementary of our review on CG-ENM).

## Discussion

### CG-beads assigned to represent molecular topology capture the slow events

The computational limitation of conventional molecular dynamics has been alleviated by coarse-grained models that reduced the number of degrees of freedom to represent biological systems in question. The mapping from all-atom to coarse-grained representation is in accord with the features of the system we choose to incorporate in the models. As always, it is a compromise between desired detail and computational efficiency. For molecular assemblies, the dynamics within each protein subunit might not be as important as those of the interactions between subunits. Thus, each subunit could be represented by a number of CG beads as long as the shape or mass distribution of the assemblies is preserved (Arkhipov et al. 2006a). C_α_-only representation of molecules has been successful in capturing slow dynamics of flap opening in HIV-1 PR (Tozzini and McCammon, 2005) and in exploring folding pathways (Clementi et al. 2000; Karanicolas and Brooks, III 2002). On the other hand, the inclusion of C_β_s is motivated to provide additional native-state constraints to limit the folding search space when C_α_ alone gives too much conformational freedom and often results in meta-stable configurations not observed in experiments (Ding et al. 2002). Here, the chemical nature of side-chains is not as much of a concern as the defined native contacts themselves although any amino-acid preference for secondary structures can be taken into account in the pseudo-dihedral potential as in the Karanicolas-Brooks model (Karanicolas and Brooks, III 2002). In the study of lipid-protein assemblies, on the other hand, the chemical nature of side-chains plays an important role and thus side-chain beads are assigned (Bond and Sansom, 2006; Shih et al. 2006).

### Effective potentials bias preferred secondary or tertiary structural features

As CG beads are being defined, effective potentials describing interactions between them have to be assigned. The choice of the potential involves the forms of the functions used for various types of interactions and the values for parameters used to define the functions. Use of more complicated functions would involve higher computation costs and difficulties in assigning their parameter values. It does seem more like art than science. By definition, Folding-based models are biased to the reference structure(s) used and therefore system specific. The potential of MM-based models are more transferable (Tozzini, 2005) and often parameterized by Boltzmann-Inversion procedure on statistical distributions of internal coordinates built on PDB structures or atomistic-MD trajectories (Arkhipov et al. 2006a). However, some weak biases are still needed to maintain preferred native features. This is illustrated by the use of C_α_ separation from a reference PDB structure as equilibrium distance in the Morse function to describe vdW interactions between alpha-carbon CG beads separated by less than 8 Å (Tozzini et al. 2007). In similar spirit, a quartic (double-well) potential for pseudo-angles is used to allow for smooth transitions between α and β secondary structures (Tozzini and McCammon, 2005; Best et al. 2005). Also, BI parameters obtained from atomistic-MD simulations of segments of all-helical apo A-I scaffold proteins properly maintained the protein secondary structures (Shih et al. 2007) that are crucial to form the correct double-belted configuration of apo A-I assembled on lipids during microsecond-long simulations (Shih et al. 2007).

The validation of CG potentials is important. Many studies have addressed the need to check the consistency of CG-MD results with those from atomistic-MD over identical simulation duration (Marrink et al. 2004; Bond and Sansom, 2006; Shih et al. 2006). For instance, Shih computed the radial distributions of mass in the satellite plant virus particle by 10-ns of CG- and atomistic-MD simulations and obtained similar profiles.

### Time-step chosen to capture the fastest motion at a given resolution

A practical aspect of CG-MD simulations is the choice of the optimal time-step, which currently seems rather *ad-hoc*. For instance, in the TTC model for HIV-1 PR dynamics, the authors used a fixed 20-fs time-step to simulate the complete catalytic cycle (Trylska et al. 2007) and an adaptive time-step that switches from 50-fs to 10-fs when dihedral or vdW energy exceeds a certain threshold to study binding pathways of ligands (Chang et al. 2007). The Hummer group used a 15-fs time-step to study sheet-helix transitions of Arc repressor (Best et al. 2005) and a 5-fs one in the calmodulin simulations (Chen and Hummer, 2007). Nevertheless, probably the largest acceptable value that still gives stable results from the Newton’s equation solver is often adopted as the optimal time-step, namely a tenth of the time-scale of the fastest motion in the system (i.e. bonds involving H-atoms vibrating at a period of 10-fs limit atomistic-MD to adopt 1-fs time-step). In Arkhipov’s virus capsid simulations, different time-steps are used for each capsid simulation. The theoretically maximal time-step in each case is related to the smallest ratio of bead mass (more accurately the reduced mass for a pair of beads) to force constant among all pseudo-bonds (Arkhipov, 2006a) since under the harmonic assumption,

$$\frac{\tau }{2\pi }=\sqrt{\frac{m}{\gamma }}$$

where *τ* is the period of oscillation for a pseudo-bond, the fastest motion in the CG system and force constant *γ* is estimated from mean-squared fluctuations of the separations between atomic-clusters (each of those mapped to a CG-bead) from an atomistic-MD simulation. The time-step used per capsid simulation ranging from 120-fs to 250-fs suggests a connection between the maximal acceptable time-step and the degree of coarse-graining (one CG bead contains ~200 atoms in this case). The combination of smaller system sizes (200 fold reduction in DOF) and larger time-steps (100 times larger than those allowed in atomistic-MD) makes CG-MD simulations of up to tens of microseconds practical.

### Solvent effect can be considered implicitly or explicitly

Representation of solvent effects is considered differently among CG-MD models. In some cases, influence of solvent on the biomolecule is ignored (Okazaki et al. 2006). When solvent effect is implicitly considered, Langevin (or Brownian) dynamics, mimicking friction and random collisions on systems in question with solvent molecules, is used (Best et al. 2005; Tozzini et al. 2007). Solvent friction was found to dampen conformational transitions (Best et al. 2005) but the slowing down of HIV-1 PR flap opening kinetics better reflects the microsecond dynamics observed in NMR (Tozzini et al. 2007) than simulations *in-vacuo*. If the real time-scale of the events is not of primary concern, a small friction or lack-of provides the advantage of faster conformational sampling.

The explicit presence of polar groups (solvent beads) drives the contacts between lipid tails and hydrophobic side-chains of proteins and therefore some models do include explicit solvent to better model the systems. For instance, the polar nature of solvent molecules is inevitably needed for lipid aggregation into correct phases (Shelley et al. 2001; Marrink et al. 2004, 2007) and for guiding the lipid-protein assembling process (Bond and Sansom, 2006; Bond et al. 2007; Shih et al. 2006, 2007). Hence the use of explicit solvent beads could be system dependent.

### The restoration of atomic details

Although more efficient conformational sampling and much longer time-scales can be attained with CG-MD, a prime limitation is that the generated conformers are devoid of atomic details. Creation of reliable and efficient methods for the reverse mapping from coarse-grained back to atomistic representation is therefore essential. Most current reconstruction of atomic details is concerned with structure prediction algorithms, the optimal configurations found from which are trained according to PDB structures (Heath et al. 2007). CG-MD structures explore a wide configurational space (including unfolded and transition states) and therefore could be different from the native (PDB) structures. Comparison of the free energy landscapes computed based on CG-MD structures and their reconstructed atomistic counterparts is proposed as a better consistency test (Heath et al. 2007). The Reconstruction Algorithm for Coarse-Grained Structures (RACOGS) proposed by the Clementi group for backbone and side-chain reconstructions was found to perform well in recovering all-atom details from CG snapshots sampled on the folding landscape (Heath et al. 2007).

### Combining the best of both worlds—multi-scale modeling

Multi-scale modeling can be done in *series* or in *parallel*. The use of atomistic-MD data to parameterize CG force-fields has been termed as the *serial multi-scale* approach by Voth and co-workers (Ayton et al. 2007). Similarly, Marrink lipid model is parameterized with alkane/water atomistic-MD simulations (Marrink et al. 2004). In these *serial* approaches, there is no direct link between atomistic and CG particles. A coupling of atomistic- and CG-models, the *parallel multi-scale* approach, would provide dynamical information across resolutions, either by embedding atomistic model in a coarse-grained model or via exchanging models at different resolutions in the spirit of Replica Exchange MD (Ayton et al. 2007). Sansom and co-workers also called for a multi-scale approach to properly model large-scale membrane dynamics while taking into account interactions of membrane proteins with their ligands (Bond et al. 2007). In order to combine genuine atomic-level details with advantages of coarse-grained simulations, a multi-scale modeling approach is well suited for further development.

### Setting up CG-MD simulations using available atomistic-MD packages

In terms of implementation, often a straightforward adaptation of existing atomistic MD packages is sufficient for the MM-based models. The TTC model for HIV-1 PR, for example, is simulated with Langevin dynamics (solutions of Newton’s equations of motion with friction and random forces included to emulate solvent effects) using the DL_POLY simulation package (Smith and Forester, 1996). In the models for virus capsid by the Schulten group, Langevin dynamics is also employed with a damping constant obtained from atomistic explicit solvent simulations of drifting protein segments (again about size of each CG bead). In order to utilize their atomistic-MD package (NAMD) with little changes and to benefit from its good performance on parallel computers, the authors adopted the potential functions defined in the CHARMM force-field for their CG-model (Arkhipov et al. 2006a). Also, little adaptations were required to implement their lipid-protein model (Shih et al. 2007).

The implementation of Folding-based models demands more effort. Wolynes and co-workers have to write their own MD code to carry out constant-temperature dynamics, without incorporating solvent effects (Okazaki et al. 2006). Hummer and co-workers applied their model to study the sheet-helix transition of an Arc Repressor mutant (Best et al. 2005). In their simulations, the single-basin Gō-potentials for the helix and sheet conformers are evaluated separately by using two copies of the CHARMM program running in parallel. The forces acting on the beads calculated for each of the single-basin Gō-potentials are then combined by weighting them with the respective Boltzmann factor. This Boltzmann-weighted force is used to evolve the protein conformation. This procedure allows helix-sheet transitions to be observed in the CG simulation (Best et al. 2005). In the subsequent application to calmodulin folding/unfolding, the minimal MD code ‘mindy’ from the NAMD package was modified and Langevin dynamics simulation was again performed (Chen and Hummer, 2007).

### Closing remarks

We have discussed CG-MD models, categorized into Folding-based and MM-based, by providing necessary physical backgrounds and exemplifying their use in the biological context. Reduced biomolecular resolution for gaining computational efficiency can be restored to atomic level with elaborate backbone/side-chain reconstructions. On the other hand, simulations at mixed/exchanged resolutions by *parallel multi-scale* approaches enrich the sampling of conformational space. However, assignment of CG beads and the choice of explicit/implicit solvent to represent specific biomolecular systems as well as biases built into the CG forcefields to favor certain secondary/tertiary structures limit the transferability of force-fields. Moreover, the choice of an optimal simulation time-step has yet to be placed on a rigorous physical basis. Nevertheless, CG-MD holds great promise in revealing functional dynamics at biologically interesting space and time-scales. Its wide-spread use is anticipated as the issues discussed above are gradually clarified.

## Figures and Tables

**Table 1:** A selection of MM-based coarse-grained models.

Model name (Reference)	CG beads per residue	Bonded			Non-bonded	
		Pseudo-bond	Pseudo-angle	Pseudo-dihedral	vdW	Electrostatics
Trylska-Tozzini- McCammon (Chang et al 2006)	on C_α_	Harmonic	Double-well*	Harmonic	Morse*	Coulomb
Schulten-SA (Arkhipov et al 2006a)	Varies	Harmonic	Harmonic	none	12–6 LJ*	Coulomb
Sansom-LP (Bond and Sansom 2006)	1 for Bb and 0–2 for Sc	Harmonic	Harmonic	None	12–6 LJ	Coulomb
Schulten-LP (Shih et al 2006)	1 for Bb and 0–1 for Sc	Harmonic	Harmonic	Cosine-series	12–6 LJ	Coulomb

* Boltzmann-Inversion parameterization employed. In Schulten-SA model, beads are assigned to groups of ~200 atoms so as to represent the shape of the protein, taking mass distribution into account. Hence, several residues are grouped into one bead.

**Abbrevations:** Lipid-protein (LP), Supramolecular-assembly (SA), Backbone (Bb), Side-chain (Sc). Note that explicit charge interactions are considered here.

**Table 2:** A selection of Folding-based coarse-grained models.

Model name (Reference)	CG beads per residue	Bonded			Non-bonded	
		Pseudo-bond	Pseudo-angle	Pseudo-dihedral	vdW	Energy landscape
Clementi-Onuchic (Clementi et al 2000)	on C_α_	Harmonic	Harmonic	Cosine-series	12–10 LJ	Single basin
Karanicolas-Brooks (Karanicolas and Brooks III 2002#)	on C_α_	Harmonic	Harmonic	Cosine-series*	12–10–6 LJ	Single basin
Wolynes-MB (Okazaki et al 2006)	on C_α_	Harmonic	Harmonic	Cosine-series	12–10 LJ	Multi-basin
Hummer-MB (Chen and Hummer 2007)	on C_α_	Constrained	Double-well*	Cosine-series*	12–10 LJ	Multi-basin
Dokholyan-DMD (Dokholyan et al 1998)	on C_α_ and C_β_	Infinite square- well	None	None	Square-well	Step-wise

* Boltzmann-Inversion parameterization employed.

# If two residues in native contact within β-sheets are H-bonded, four additional weak interactions (at 1/4 strength of the H-bond) among neighboring beads are assigned to maintain the backbone configuration.

**Abbreviations:** MB: Multiple-basin; DMD: Discrete Molecular Dynamics.

## Abbreviations

MD: Molecular Dynamics
ENM: Elastic Network Model
DOF: Degree Of Freedom
LJ: Lennard-Jones
PDB: Protein Data Bank
